# Demonstration of Monolithic Integration of InAs Quantum Dot Microdisk Light Emitters and Photodetectors Directly Grown on On-Axis Silicon (001)

**DOI:** 10.3390/mi16080897

**Published:** 2025-07-31

**Authors:** Shuaicheng Liu, Hao Liu, Jihong Ye, Hao Zhai, Weihong Xiong, Yisu Yang, Jun Wang, Qi Wang, Yongqing Huang, Xiaomin Ren

**Affiliations:** State Key Laboratory of Information Photonics and Optical Communications, Beijing University of Posts and Telecommunications, Beijing 100876, China; liusc@bupt.edu.cn (S.L.); jhye@bupt.edu.cn (J.Y.); zhaihao@bupt.edu.cn (H.Z.); xwh@bupt.edu.cn (W.X.); yangyisu@bupt.edu.cn (Y.Y.); wangjun12@bupt.edu.cn (J.W.); wangqi@bupt.edu.cn (Q.W.); yqhuang@bupt.edu.cn (Y.H.); xmren@bupt.edu.cn (X.R.)

**Keywords:** silicon photonic chips, monolithic integration, heteroepitaxial growth, quantum dot, microdisk light emitter, photodetector

## Abstract

Silicon-based microcavity quantum dot lasers are attractive candidates for on-chip light sources in photonic integrated circuits due to their small size, low power consumption, and compatibility with silicon photonic platforms. However, integrating components like quantum dot lasers and photodetectors on a single chip remains challenging due to material compatibility issues and mode field mismatch problems. In this work, we have demonstrated monolithic integration of an InAs quantum dot microdisk light emitter, waveguide, and photodetector on a silicon platform using a shared epitaxial structure. The photodetector successfully monitored variations in light emitter output power, experimentally proving the feasibility of this integrated scheme. This work represents a key step toward multifunctional integrated photonic systems. Future efforts will focus on enhancing the light emitter output power, improving waveguide efficiency, and scaling up the integration density for advanced applications in optical communication.

## 1. Introduction

Silicon photonic integration is characterized by the monolithic integration of photonic and electronic components on a silicon substrate. This technology is compatible with a complementary metal oxide semiconductor (CMOS) infrastructure, allowing it to continue leveraging the advantages of a CMOS for large-scale integration and ultrahigh-precision manufacturing. It fully utilizes the transmission advantages of photons as information carriers, such as high speed, a large capacity, low crosstalk, and anti-interference capabilities, thereby enhancing the overall performance of chips without the need to pursue extreme device size reduction. This significantly reduces the difficulty of manufacturing processes, making it a core technology in the post-Moore era [1,2,3]. With the maturation and continuous development of silicon photonics technology, low-loss silicon optical waveguides, passive silicon photonic devices such as multiplexers and demultiplexers, and high-speed silicon optical modulators have enabled large-scale silicon-based monolithic photonic integration [4]. Meanwhile, mature integration schemes for photodetectors have been demonstrated [5,6]. However, silicon is an indirect bandgap material, making it challenging to achieve high-efficiency luminescence in pure silicon material devices. There is still a lack of mature solutions for the large-scale production of silicon-based integrated light sources.

Looking forward, the direct growth of III–V compound materials on silicon substrates and the fabrication of lasers through monolithic integration are more promising for achieving low-cost, large-scale, and multi-wavelength silicon photonic chips [7]. Researchers have currently developed silicon-based III–V semiconductor lasers and other on-chip light sources through hybrid integration [8], heterogeneous integration [9], and monolithic integration [10], actively exploring their applications in silicon photonic chips. Monolithic integration involves the use of silicon-based III–V semiconductor heteroepitaxy growth technology to directly grow III–V semiconductor materials and laser structures on silicon substrates or virtual substrates such as SOI [11], Ge/Si [12], GaP/Si [13], and GaAs/Si [14], without the need for III–V substrates. In the long term, this approach offers high integration density and scalability advantages, making it promising for achieving low-cost, large-scale, multi-wavelength silicon photonic chips [15]. Leveraging the advantages of quantum dot (QD) materials, particularly their three-dimensional quantum confinement effects, InAs QD lasers exhibit superior photonic properties characterized by an ultralow-threshold current density [16], high temperature stability [17], strong tolerance to external feedback [18], and low relative intensity noise [19]. These features lead to improved power efficiency and a significant reduction in noise levels, which play a crucial role in enhancing the overall performance of integrated photonic devices [20,21,22,23]. In addition, the monolithic integration of InAs QD lasers on silicon benefits from its tolerance to material defects, achieving a room-temperature operational lifetime of millions of hours [24], which meets the practical requirements. However, only a limited number of theoretical simulations or experimental demonstrations have been reported regarding the monolithic integration of silicon-based heteroepitaxial QD lasers with waveguides [25,26,27,28,29]. Furthermore, no on-chip high-speed optical transmission chips incorporating such heteroepitaxial III–V quantum dot lasers have been reported to date. The approach involving full monolithic integration of all optoelectronic devices through heteroepitaxial techniques remains far from practical implementation.

Although the Fabry–Perot cavity InAs QD laser selectively grown on patterned silicon substrates has achieved monolithic integration with silicon waveguides [29,30], it requires precise alignment during the growth process to align the active region with the silicon waveguide. The III–V compound semiconductor materials grown in the patterned windows tend to transform into polycrystalline structures at the boundaries, leading to numerous material defects. It is necessary to use focused ion beam (FIB) etching to remove these defective materials and simultaneously etch the cavity surfaces required for the Fabry–Perot cavity InAs QD lasers. This complex fabrication process, which relies on time-consuming FIB etching that may damage laser facets, results in a significantly reduced yield and substantially increased production costs. Achieving multi-wavelength lasers on silicon substrates based on this approach is challenging [31]. In comparison, silicon-based whispering gallery mode (WGM) optical microcavity QD lasers offer several advantages as on-chip light sources [32,33,34,35,36]. Firstly, by altering the geometrical parameters of the microcavity, such as the diameter of the microdisks, and introducing various on-chip tuning mechanisms, the cavity modes of the microcavity can be flexibly adjusted. This makes it relatively easy to achieve multi-wavelength light sources on silicon-based chips through multiple microcavities. Secondly, microcavity lasers have the advantages of a smaller size, higher quality factor, lower threshold current, and lower power consumption, making them more suitable as on-chip light sources for large-scale silicon photonic chips. Thirdly, the III–V epitaxial structure of the microcavity lasers can be shared with other optoelectronic devices or passive optical waveguides. This directly eliminates the stringent requirements in silicon photonic integration, such as anti-reflection at the facets, facet alignment, and facet coupling. Moreover, the materials, structures, and processes involved in photonic integration are perfectly compatible, which can simplify and facilitate silicon-based monolithic photonic integration technology.

In this work, by fabricating microdisk light emitters, waveguides, and photodetectors from an identical epitaxial material structure, we demonstrate a preliminary silicon-based monolithically integrated photonic chip. The integrated photodetectors successfully detect the output power of the microdisk light emitters, thereby validating the functional compatibility of the shared material platform. This approach establishes a scheme for monolithic integration in silicon photonics, providing critical insights into the development of scalable on-chip light sources with potential applications in next-generation optoelectronic systems.

## 2. Growth and Fabrication

**Figure 1:**
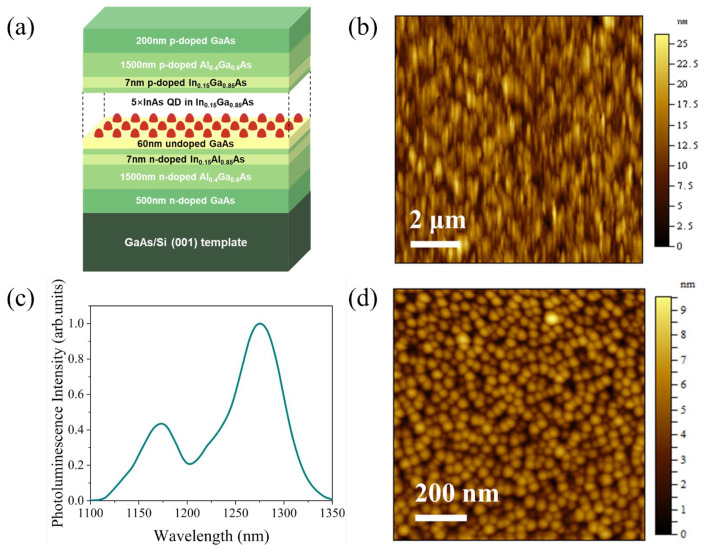
(**a**) Schematic of the layer structure. (**b**) AFM image (10 μm × 10 μm) of 1000 nm GaAs buffers grown on on-axis Si (001) substrate with a root mean square roughness of 3.27 nm. (**c**) Room-temperature photoluminescence spectrum of the QD active region grown on the Si substrate. (**d**) AFM image (1 μm × 1 μm) of QDs with a density of 5.2 × 10^10^/cm^2^.

The epitaxial structure of the microdisk light emitters, as well as the waveguides and photodetectors, was directly grown on the on-axis Si (001) substrate using a solid-source molecular beam epitaxy system, and the schematic of the structure is shown in Figure 1a. We initiated growth on the GaAs/Si (001) template without antiphase domains (APDs). More information about the virtual substrate growth conditions can be found in Ref. [37]. The surface morphology of the GaAs/Si (001) template was measured by atomic force microscopy (AFM), as shown in Figure 1b. There was no APD on the sample surface, and the root mean square roughness was 3.27 nm (10 μm × 10 μm).

For the growth of the microdisk light emitter structure, a 500 nm GaAs *n*-contact layer and a 1500 nm Al_0.4_Ga_0.6_As lower *n*-cladding layer were grown on the aforementioned virtual substrate, with Si-doping concentrations of 4 × 10^18^/cm^3^ and 0.5~1 × 10^18^/cm^3^, respectively. Then, a 60 nm undoped GaAs layer and five periods of InAs QDs in a 5 nm In_0.15_Ga_0.85_As quantum well separated by 45 nm GaAs spacer layers were grown as the active region. The detailed process of the structure’s growth can be found by referring to our previous report [14]. For the room-temperature photoluminescence (PL) spectrum of the sample, the peak wavelength and full-width at half-maximum (FWHM) are 1274 nm and 43 meV, as shown in Figure 1c. Figure 1d shows the AFM image of the uncapped QDs under the same growth conditions, where the QD density is about 5.2 × 10^10^/cm^2^. Afterward, 1500 nm Al_0.4_Ga_0.6_As and 200 nm GaAs layers were grown as *p*-cladding and *p*-contact layers, with Be-doping concentrations of 0.5~1 × 10^18^/cm^3^ and 2 × 10^19^/cm^3^, respectively. Additionally, 7 nm *p*-doped In_0.15_Ga_0.85_As and 7 nm *n*-doped In_0.15_Al_0.85_As layers were grown and inserted as gliding dislocation displacing layers at a distance of 50 nm above and below the active region, which can displace the gliding dislocation from the active region [38,39].

**Figure 2:**
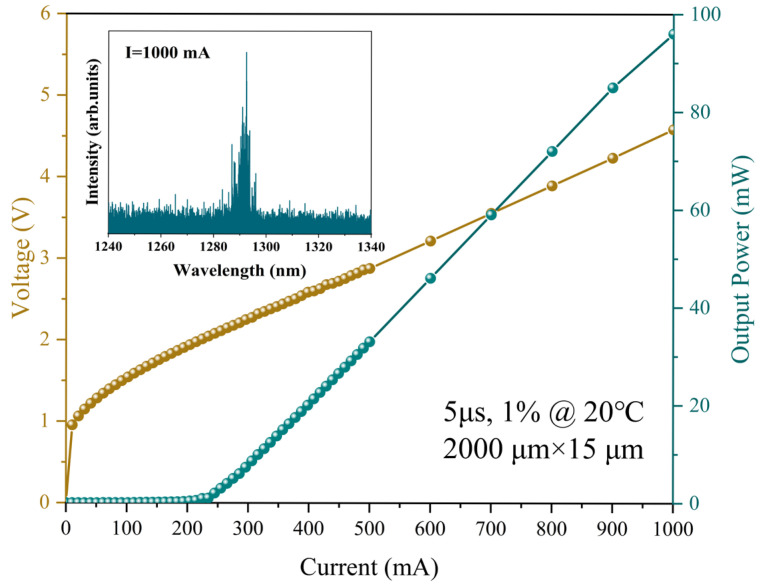
P-I–V characteristics for a 15 μm × 2 mm edge-emitting QD laser grown on a GaAs/Si (001) template under pulsed conditions at room temperature. The inset shows the lasing spectrum of the laser at an injection current of 1000 mA.

To evaluate the performance of InAs QD material directly grown on the GaAs/Si (001) template, the heterostructure was processed into broad-stripe lasers with as-cleaved facets and ridges of 15 μm in width and 2 mm in length. No facet coating was applied. The devices were probed under pulsed conditions at room temperature (20 °C). Figure 2 shows the typical output power–current–voltage (P–I–V) characteristics for the QD laser grown directly on the GaAs/Si (001) template. The measurements were performed under pulsed current conditions with a pulse width of 5 μs and a duty cycle of 1%. The device exhibits a series resistance of 3 Ω and a turn-on voltage of 0.95 V. The threshold current of the laser was 263 mA, corresponding to a threshold current density of 877 A/cm^2^. When the injection current was 1000 mA, the single-facet peak power and the slope efficiency were 96 mW and 0.128 W/A, respectively, with no evidence of power saturation up to this current. The light emission spectrum measured above the threshold current (1000 mA) reveals the lasing wavelength of 1292.5 nm.

**Figure 3:**
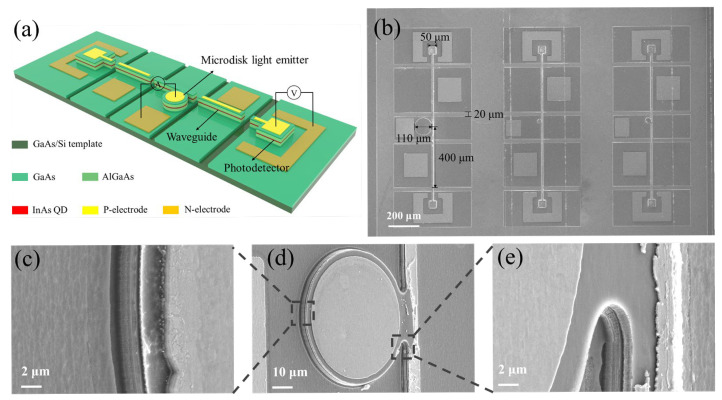
(**a**) Schematic diagram of the silicon-based optoelectronic integrated system. (**b**) SEM image of the silicon-based optoelectronic integrated system. (**c**) SEM image of the microdisk light emitter sidewall. (**d**) SEM image of the relative positions of the microdisk light emitter and the waveguide. (**e**) SEM image of the tangent region between the microdisk light emitter and the waveguide.

Subsequently, the same wafer was processed by standard photolithography and inductively coupled plasma (ICP) etching to define the microdisk light emitter and photodetector structures. Firstly, Ti/Pt/Au (50/50/300 nm) films were evaporated by electron beam evaporation as the *p*-type metal electrodes. Subsequently, microdisk light emitters coupled with a 10 μm wide waveguide and photodetectors were defined using ICP etching down to the *n*-contact GaAs layer. Next, to prevent current from flowing between different devices, an additional deep etching was performed using ICP until reaching the undoped layer of the GaAs/Si template, thereby creating insulating channels between the waveguide and the active devices. Afterwards, AuGe/Ni/Au (50/25/300 nm) films were evaporated by electron beam evaporation as the *n*-type metal electrodes. No sidewall coating or passivation was applied. Figure 3a presents the schematic diagram of the silicon-based optoelectronic integrated system. The scanning electron microscope (SEM) image of the silicon-based optoelectronic integrated system is shown in Figure 3b. A typical microdisk light emitter has a diameter of 110 μm, and the total length of the waveguide is approximately 400 μm. The photodetector is designed with a side length of 50 μm. The insulating channel width between the devices is 20 μm. Figure 3c–e present locally magnified images of the microdisk light emitter sidewall and the tangent region between the microdisk light emitter and the waveguide, illustrating the slope and surface roughness that can influence optical losses.

## 3. Results and Discussion

**Figure 4:**
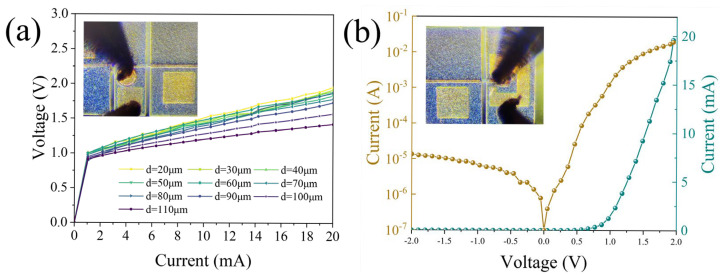
(**a**) I–V characteristic curve of the microdisk light emitter. (**b**) I–V characteristic curve of the photodetector plotted in the linear and logarithmic coordinates, respectively.

The current–voltage (I–V) characteristics of microdisk light emitter with different diameters measured at 15 °C under continuous-wave (CW) operation are shown in Figure 4a. As the diameter of the microdisk light emitters increases from 20 μm to 110 μm, the corresponding differential resistance decreases from 43 Ω to 23 Ω. Figure 4b illustrates the I–V curves of the photodetector in both linear and logarithmic scales under dark conditions. At zero-bias voltage, the dark current is 0.4 μA and the resistance is 62 Ω. The rectification ratio of the photodetector is approximately 1465 within the voltage range of ±2 V.

**Figure 5:**
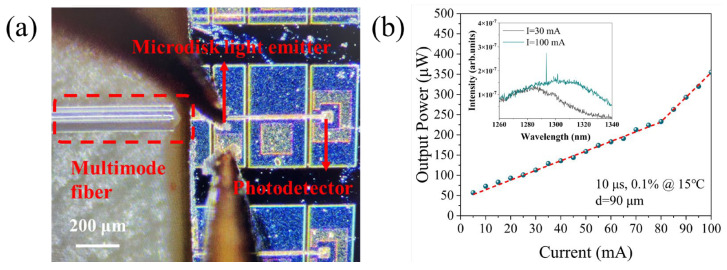
(**a**) Schematic illustration of optical fiber collecting light from the waveguide during testing. (**b**) P-I characteristics of the waveguide output power versus injection current for the microdisk light emitter. The inset shows the fluorescence spectrum at 30 mA and the lasing spectrum at 100 mA under pulsed operation.

After measuring the I–V characteristics, we proceeded to test the output power of the microdisk light emitter. To this end, the waveguide was cleaved near its midpoint to measure the light coupled from the microdisk light emitter into the waveguide. Figure 5a illustrates the optical fiber aligned to the waveguide for collecting the light during the measurement. We tested a representative microdisk light emitter with a diameter of 90 μm. The output power was first measured using an integrating sphere, followed by spectral measurements using a multimode fiber. All measurements were performed under pulsed conditions with a 10 μs pulse width and 0.1% duty cycle. The output power–current (P-I) characteristics of the microdisk light emitters are presented in Figure 5b, with the inset showing the fluorescence spectrum at 30 mA and the lasing spectrum at 100 mA, where the lasing wavelength is 1293 nm.

**Figure 6:**
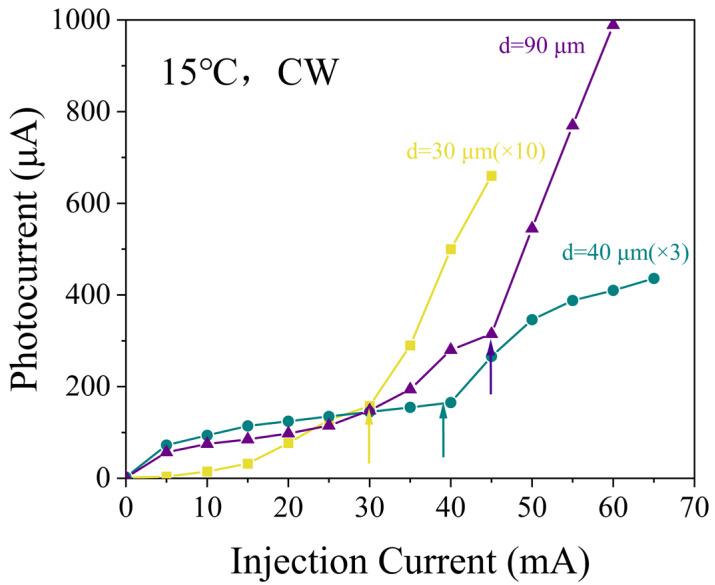
Photocurrent as a function of injection current for microdisk light emitters of various integrated systems.

To evaluate the integration performance of the microdisk light emitter and photodetector, the photodetector was biased at −1 V, while the microdisk light emitter was operated under CW conditions. Before the current was injected into the microdisk light emitter, the photodetector exhibited a response current of a few microamperes, corresponding to its dark current. Specifically, the dark currents for photodetectors integrated with microdisk light emitters of 30 μm, 40 μm, and 90 μm diameters were measured to be 1.2 μA, 2.66 μA, and 1.7 μA, respectively. Upon injecting current into the microdisk light emitter, the photodetector began to exhibit a response current that exceeded the dark current by more than an order of magnitude. As the injection current increased, the photocurrent initially increased gradually and then exhibited a steep increase, consistent with the threshold behavior typically observed of the lasers. Figure 6 presents the original data for three representative integrated systems, showing the relationship between the photodetector response current and the injection current of the microdisk light emitters. For devices with diameters of 30, 40, and 90 μm, we observe a distinct kink in the curve at approximately 30, 38, and 45 mA, respectively. Beyond this point, the photocurrent exhibits a significantly steeper increase with injection current. The corresponding current densities at these kink points are calculated to be 4244, 3024, and 707 A/cm^2^, respectively. This observed change in slope is a characteristic feature often associated with the transition towards stimulated emission. However, the precise nature of this transition under CW conditions requires further spectral characterization.

The results indicate that the current density at the kink point is higher for smaller-diameter microdisk light emitters. This phenomenon arises from increased optical losses in microdisk light emitters, primarily originating from three sources [32,33,34,35,36,40]. First, light absorption in the near-surface region plays a dominant role, associated with non-radiative recombination at the microdisk surface. Second, curvature-induced optical losses occur due to the cylindrical cavity geometry, where smaller microdisk light emitters experience greater optical mode leakage, leading to enhanced radiative losses. Third, light scattering is caused by sidewall roughness. The higher surface-to-volume ratio in compact microdisk light emitters increases the probability of light interacting with sidewall imperfections, thereby amplifying scattering losses. Consequently, these combined effects result in elevated threshold current densities for such devices. To address these losses, future work will focus on optimizing the gap between the microdisk and waveguide to improve coupling efficiency, as well as refining fabrication processes to smooth sidewalls and reduce scattering.

**Figure 7:**
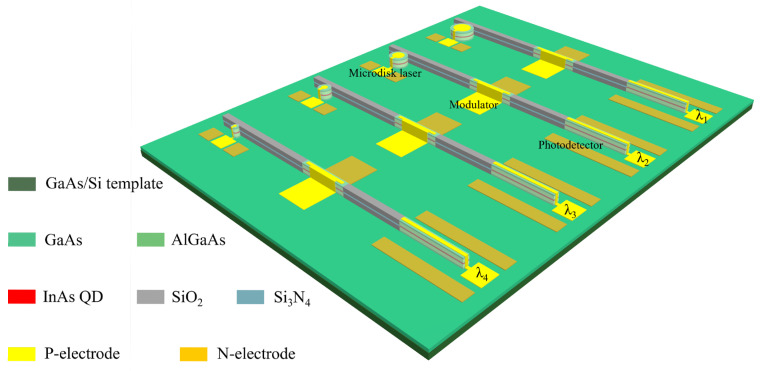
Schematic diagram of the microlaser integration on a silicon platform.

To enable advanced functionality, an electrode structure can be monolithically integrated onto the waveguide. Under forward bias, optical amplification is achieved through carrier injection, whereas reverse bias enhances waveguide losses, resulting in effective optical attenuation. This mechanism enables laser modulation, thereby facilitating the development of a prototype for silicon photonic chips. In addition to varying microcavity dimensions, multiple on-chip tuning methods such as electro-optic and thermo-optic tuning can be employed to achieve flexible multi-wavelength operation within the microdisk laser arrays. However, scaling integration density poses challenges such as optical crosstalk and heat accumulation. Optimized waveguide design and thermal management are essential for high-density, scalable photonic integration. This work proposes an approach to integrating microdisk laser arrays on silicon photonic integrated circuits, as schematically illustrated in Figure 7.

## 4. Conclusions

In conclusion, we have demonstrated the monolithic integration of functional photonic components, including microdisk light emitters, waveguides, and photodetectors, fabricated from a shared epitaxial structure on a silicon platform. This material platform not only simplifies the fabrication process but also ensures optimal compatibility and performance across all integrated devices. In the future, we will further enhance the optical gain of QDs while optimizing the structures of waveguides and photodetectors to achieve more effective laser modulation, thereby enabling more efficient information transmission. The ultimate goal is the realization of multi-wavelength, silicon-based monolithic photonic integrated circuits. Such a breakthrough holds significant implications for the development of next-generation silicon photonic integrated circuits.

## Data Availability

The original contributions presented in the study are included in the article, further inquiries can be directed to the corresponding author/s.
